# Herbicides Have Minimal and Variable Effects on the Structure and Function of Bacterial Communities in Agricultural Soils

**DOI:** 10.1111/1462-2920.70148

**Published:** 2025-07-14

**Authors:** Margaret Gaylord, Abigail Thompson, Franck E. Dayan, Andrew R. Kniss, Dave Reichert, Kristen Otto, Rebecca Larson, Pankaj Trivedi

**Affiliations:** ^1^ Microbiome Network Colorado State University Fort Collins Colorado USA; ^2^ Department of Agricultural Biology Colorado State University Fort Collins Colorado USA; ^3^ Department of Plant Sciences University of Wyoming Laramie Wyoming USA; ^4^ Western Sugar Cooperative Scottsbluff Nebraska USA; ^5^ Western Sugar Cooperative Denver Colorado USA; ^6^ Institute of Genomics for Crop Abiotic Stress Tolerance (IGCAST), Department of Plant and Soil Science Texas Tech University Lubbock Texas USA

## Abstract

Synthetic herbicides are relied on for weed management in the United States, and non‐chemical alternatives, such as tillage, can be inefficient and expensive at an agricultural scale. Previous research examining the influence of weed management strategies on soil microbial communities has shown variability and inconsistency in experimental design and results. This study investigates the impact of repeated applications of glyphosate, a mixture of selective herbicides, and tillage on soil bacterial communities in sugar beet production across two locations in the United States. Using next‐generation sequencing and various assays, we explore the effects on bacterial community structure, composition, and function related to nutrient cycling and soil health. Although transient and minor differences in bacterial community structure could be observed for herbicides and tillage, these statistical differences do not appear to be biologically relevant as the soil function was equivalent for all applied treatments. Our findings provide evidence that repeated herbicide usage does not directly impact soil health and function in sugar beet production. This study emphasises the need for well‐replicated, field‐realistic, and long‐term experiments to understand the ecological consequences of herbicides and tillage in agroecosystems.

## Graphical abstract

In vitro analysis of glyphosate impacts does not reflect dynamics in the agroecosystem. This study indicates the importance of a diverse, cooperative microbiome in field soils that could improve resilience of the community that cannot be achieved in a laboratory setting.

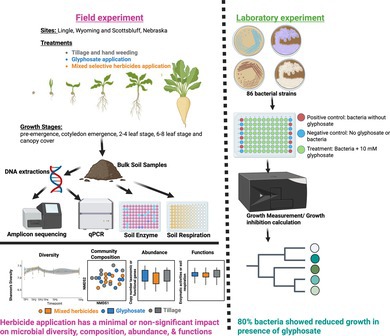

## 1 Introduction

Weed pressure in the United States is managed with herbicides, mechanical removal (tillage), or a combination of the two. Arguably, of these options, synthetic herbicides have afforded the most predictable and complete weed control. At scale, tillage can be inefficient, inefficient, and expensive. Even with skilled operators, tillage can directly cause crop damage and incomplete weed control leads to unrecognised crop yield potential (Machleb et al. 2020; Monteiro and Santos 2022). Tillage can also have the deleterious side effect of promoting further weed pressure, increasing greenhouse gas emissions associated with agricultural production, and reducing soil health (Cioni and Maines 2010; Kraut‐Cohen et al. 2020).

It is estimated that 87 million hectares of agricultural land in the United States are treated with herbicides annually to avoid $15.5 billion in revenue losses from weed competition (Gianessi and Reigner 2007). Herbicides are classified based on their selectivity, mode of action, and translocation in the plant (Duke and Dayan 2022; Vats 2015). Careful timing and selection of selective herbicides are necessary to gain proper control throughout the agricultural season (Bhadra et al. 2020; Carey and Kells 1995; Jursík et al. 2008). Furthermore, based on the selective nature, the application of multiple products is typically necessary to control the broad spectrum of weed species (Beffa et al. 2019; Dayan 2019; Morishita 2018) and prevent herbicide resistance development within weed populations (Mehdizadeh et al. 2022; Nakka et al. 2019). Due to their popularity and increased use in agricultural systems, the impacts of continued use of these chemicals on human and animal health, non‐target plants, microorganisms, and the environment have been an important debate (Kniss 2017; Puigbò et al. 2022; Ruuskanen et al. 2023).

Soil microbiome plays a critical role in soil functioning and is essential for plant growth and health (Singh et al. 2020; Trivedi et al. 2021). Tillage is well documented to have a large negative effect on soil microbiome health, function, and diversity (Farmaha et al. 2022; Kraut‐Cohen et al. 2020; Nunes et al. 2020; Veum et al. 2022). Likewise, herbicides are postulated to impact ecosystem functions via altering soil microbiome either directly by impacting survival or function (Chávez‐Ortiz et al. 2022; Darine et al. 2015; Valle et al. 2006) or indirectly by altering host function (Fuchs et al. 2021; Lu et al. 2019; Mohamed et al. 2021). For example, some herbicides can have a negative impact on microbial community structure and functioning (Cycoń et al. 2012; Cycoń et al. 2013; Newman et al. 2016; Tanney and Hutchison 2010; Zobiole et al. 2011). However, the effects are unlikely biologically relevant since they are rate‐dependent, and most studies indicate that when applied at or below their recommended field‐rate, herbicides exert negligible (Barriuso et al. 2011; Lane et al. 2012; Ratcliff et al. 2006; Rosenbaum et al. 2014) or minor (Barriuso et al. 2010; Barriuso and Mellado 2012; Weaver et al. 2007) effects on microbial community structure, and negligible effects on functionality (Ratcliff et al. 2006). Inconsistent results are not surprising given the strong influence of location and soil characteristics on microbial communities and the generally small effect of herbicide treatments at a field scale. Moreover, the limited resolution of the techniques used to determine the impacts of herbicides on microbial community composition or diversity may have contributed to the variability (Arango et al. 2014; Newman et al. 2016; Tanney and Hutchison 2010).

Sugar beet is an economically important crop that accounts for almost 50% of the sugar in the United States, and about 30% worldwide (Barker and Dayan 2019; Bhadra et al. 2020). Weed control is an essential cultural management strategy for sugar beet production. Without it, yield losses could reach up to 83%, leading to a monetary loss of approximately $1.25 billion for US growers (Soltani et al. 2018). As a poorly competitive crop, sugar beet emerges slowly and has delayed canopy development; chemical application programmes are necessary from crop emergence until the eight‐leaf stage (Gerhards et al. 2017; Hoffmann et al. 2021). The use of the non‐selective herbicide glyphosate has increased dramatically since the wide acceptance of genetically modified glyphosate‐resistant sugar beet (Kniss 2010; Stevanato et al. 2019). Repeated applications are required to control diverse weed species germinating throughout the growing season (Jursík et al. 2008). Several studies have reported the importance of soil microbiota in promoting disease resistance against a range of sugar beet pathogens (e.g., *Rhizoctonia solani, Fusarium* sp., and *Pythium* sp.) and sustaining sugar beet yields via improved nutrient uptake and stress tolerance (Carrión et al. 2018; Carrión et al. 2019; Chapelle et al. 2016; Du et al. 2022; Mendes et al. 2011; Postma and Schilder 2015; Watanabe et al. 2011). In light of the importance of the soil microbiome, the potential consequences of various weed management strategies on the microbial structure and function for sugar beet could impact cultural management strategies significantly. However, there is no report on the effects of weed management strategies on soil microbial community and functioning in sugar beet production using well‐replicated, properly controlled field studies at different locations.

The soil microbiota are central to soil health; therefore, protecting the diversity and function of the soil microbiome is critical for sustainable farming. Weeds are an omnipresent threat for all agricultural producers. Failure to manage weeds results in significant competition and unrecognised yield potential, which is the number one driver of biodiversity loss worldwide (Willett et al. 2019). To date, most studies focus on the impact of one weed management strategy or another on soil health. This research aims to contrast the impact of repeated glyphosate applications, a mixture of alternative selective herbicides registered for use on sugar beets, and tillage for weed management on soil bacterial communities in sugar beet production under field conditions over two locations. This study also seeks to evaluate whether in vitro studies are informative of impacts in the agroecosystem. Using amplicon sequencing, we first explored the impact of herbicide application and tillage on the structure and composition of soil bacterial communities. We then measured soil enzymatic activities, microbial respiration, and abundances of several genes involved in nutrient cycling to evaluate the impact of herbicides and tillage on microbial‐mediated functions. We further performed in vitro assays to assess the effect of glyphosate application on the growth of soil bacterial isolates.

## 2 Materials and Methods

### 2.1 Experiment Design

Two field experiments in sugar beet plots were established at Lingle, Wyoming, and Scottsbluff, Nebraska. Our sites varied significantly in terms of basic soil properties and nutrient contents (Table S1). In each site, 12 experimental plots were established in four replicate blocks with three treatments randomly allocated to plots in each block. A detailed description of the herbicide rates for the glyphosate, mixed selective herbicides, and the tillage treatment that serves as an experimental control treatment is available in Table 1. For the tillage treatment, soil between the sugar beet rows was tilled using a cultivators with sweeps set to a 7‐cm soil depth. Both sites selected for this study have been managed with conventional tillage outside of this study. Glyphosate was applied at the 2 true‐leaf, 6–8 true‐leaf, and canopy closure growth stages. The mixed selective herbicide treatments consisted of the application of a pre‐emergence herbicide (ethofumesate) followed by three post‐emergence applications (phenmedipham + desmedipham + triflusulfuron + clopyralid) at the cotyledon, 2 true‐leaf, and 4–6 true‐leaf growth stages. The tillage treatment plots were tilled and hand‐weeded twice, at the 4–6 true‐leaf and 6–8 true‐leaf growth stages. As 100% of sugar beet hectares in North America are sprayed with herbicides or tilled for weed control (Soltani et al. 2018), our experimental design compares the effects of realistic weed management alternatives on the structure and function of soil microbial communities.

**TABLE 1:** Summary of the experimental design used in this study.

Timings	Products	Active ingredient or method	Formulation	Rate	
Glyphosate application					
2 True‐leaf	Roundup PowerMax	Glyphosate, potassium salt	SL	1.5 kg a.e./ha	
6–8 True‐leaf	Roundup PowerMax	Glyphosate, potassium salt	SL	1.5 kg a.e./ha	
Canopy	Roundup PowerMax	Glyphosate, potassium salt	SL	1.5 kg a.e./ha	
Mixed selective herbicides application^a^					
Preemergence	Nortron	Ethofumesate	SC	1.1 kg/ha	
Cotyledon	Betamix	Phenmedipham + desmedipham	EC	92 + 92 g a.i./ha	
	Upbeet	Triflusulfuron methyl	DF	8.8 g a.i./ha	
	Stinger	Clopyralid, monoethanolamine salt	SL	63 g a.e./ha	
2 True‐leaf	Betamix	Phenmedipham + desmedipham	EC	92 + 92 g a.i./ha	
	Upbeet	Triflusulfuron methyl	DF	8.8 g a.i./ha	
	Stinger	Clopyralid, monoethanolamine salt	SL	63 g a.e./ha	
4–6 True‐leaf	Betamix	Phenmedipham + desmedipham	EC	92 + 92 g a.i./ha	
	Upbeet	Triflusulfuron methyl	DF	8.8 g a.i./ha	
	Stinger	Clopyralid, monoethanolamine salt	SL	63 g a.e./ha	
	Warrant	Acetochlor	CS	1 kg a.i./ha	
Cultivation only					
4–6 True‐leaf		Tillage			
		Handweeding			
6–8 True‐leaf		Tillage			
		Handweeding			

Abbreviations: CS = micro‐encapsulated suspension; DF = dry flowable; EC = emulsifiable concentrate; SC = suspension concentrate; SL = soluble liquid concentrate; a.e., acid equivalent [Glyphosate can be formulated as different salts (potassium salt, isopropyl amine salt, others), and the glyphoste‐salt is the ‘active ingredient’. To standardize glyphosate rates across formulations, we have listed the rate as glyphosate acid equivalent]; a.i., active ingredient.

^a^ Methylated seed oil (MSO) was added to the mixed selective herbicide treatments at 1.5% v/v.

### 2.2 Sample Collection and DNA Extraction

Samples were collected at the same six timepoints for all treatments, 3–4 days after each herbicide application. Bulk soil samples were collected at pre‐emergence, cotyledon emergence, 2–4 leaf stage, 6–8 leaf stage and canopy closure. The six sampling timepoints were uniform across all the treatments. At each sampling timepoint, we combined three soil cores per plot taken at a depth of 0–7.5 cm. Samples from each plot were mixed thoroughly and shipped on ice to Colorado State University. Field moist soil was immediately passed through a 5.6 mm sieve for biological analysis and air‐dried and sieved (< 2 mm) for chemical analysis. Aliquots of soil samples were stored at −80°C for subsequent DNA extraction. DNA was extracted from soils using the DNeasy PowerSoil Pro Kit (QIAGEN, Hilden, Germany) as per the manufacturer's instructions. Extracted DNA was quality checked by NanoDrop 2000 (Thermo Fisher Scientific, Waltham, Massachusetts, USA) and quantity checked by Qubit Fluorometer (Thermo Fisher Scientific) using Quant‐iT dsDNA BR Assay Kit (Invitrogen, Carlsbad, CA, USA), and stored at −80°C.

### 2.3 Amplicon Sequencing and Bioinformatic Analyses

Bacterial 16S rRNA gene V4‐V5 hypervariable regions were PCR amplified using primers 515F/806R (Caporaso et al. 2012) combined with adapter sequences and barcode sequences. Purified amplicons were sequenced at the Next Generation Sequencing Facility, Colorado State University on an Illumina MiSeq platform (Illumina Inc., San Diego, CA). Bioinformatics processing was performed using a combination of USEARCH (Edgar 2010) and UNOISE3 (Edgar 2016). Operating taxonomic unit (OTU) tables based on 97% sequence similarity were generated using the USEARCH pipeline. Sequencing run quality was assessed using fastQC (Andrews 2010). The raw sequences were discarded if they contained ambiguous nucleotides, had a low (*Q* < 20) quality score, or were short in length (< 100 bp). Adapters and primers were removed using cutadapt (Martin 2011). Then, samples were demultiplexed. Paired‐end reads were merged, and quality was assessed with an initial quality check test. The representative set database was created using the UPARSE algorithm (Edgar 2013). Unique sequences were located and sorted into unique OTUs. OTUs were clustered using DADA2 and de‐noised using uNoise3 (Xiong, He, et al. 2021) as described in Xiong, Zhu, et al. (2021). OTU tables were generated by mapping reads to the representative set database. OTUs were counted at the sample level. Taxonomic identification of bacteria was obtained against the Silva database (Pruesse et al. 2007). Bacterial sequences that matched host mitochondria and chloroplast were removed.

### 2.4 Soil Enzymatic Activities

β‐d‐cellobiohydrolase (CB), α‐glucosidase (AG), β‐glucosidase (BG), *N*‐acetyl‐β‐glucosaminidase (NAG), phosphatase (PHOS) and β‐xylosidase (XYL) activities were measured using 4‐methylumbelliferyl‐β‐d‐galactoside (MUB‐gal) substrate, yielding the highly fluorescent cleavage products MUB upon hydrolysis. ΑG, BG, XYL, and CB contribute to carbon cycling and plant litter decomposition, which further promotes the growth and activity of soil bacteria (Adetunji et al. 2017; Fan et al. 2012; Matsuzawa et al. 2016; Štursová and Baldrian 2011). PHOS mineralises organic phosphorous into a plant‐available form (Nannipieri et al. 2011) and NAG is involved in nitrogen cycling (Kang et al. 2005). All the enzyme assays were set up in 96‐well microplates as described by Bell et al. (2013). Twelve replicate wells were set up for each sample and each standard concentration. The assay plate was incubated in the dark at 25°C for 3 h to mimic the average soil temperature. Enzyme activities were corrected using a quench control. Fluorescence was measured using a Tecan Infinite M200 (Tecan, Mannedorf, Switzerland) with 365‐nm excitation and 460‐nm emission filters. The activities were expressed as nmol h^−1^ g^−1^ dry soil. Enzyme activities were calculated for soil samples from two sites collected at all the time points.

### 2.5 Soil Respiration Analysis

Soil respiration was measured using MicroResp (Macaulay Scientific Consulting, UK). Approximately 350 mg of soil was added to deep well microtitre plates to which 30 μL of water was added in each well. A rubber sealing mat was used to seal the deep well plate to an indicator plate, and plates were incubated in the dark over 6 h at 25°C as previously. After incubation, the CO_2_ production rate was calculated based on the change in absorbance (A_570_) of the indicator plate (Campbell et al. 2003).

### 2.6 Quantitative Real‐Time PCR (qPCR) Analysis

qPCR was performed to determine gene copy numbers for total bacteria and functional genes involved in nitrogen fixation (*nifH* encoding dinitrogenase reductase); denitrification (nirK and nirS encoding the cd1 and copper nitrite reductase; nosZ encoding the nitrous oxide reductase); phosphorous mineralisation (phoC encoding for acid phosphatase); cellulose degradation (GH11 encoding for xylanase); and chitin degradation (GH18 encoding for chitinase). qPCR was conducted using primers and cycling conditions described previously (Fraser et al. 2017; Trivedi et al. 2012; Wang et al. 2022). qPCR reactions were carried out on extracted soil DNA from timepoints 1, 3, and 6 using Bioline SensiFAST qPCR SYBR green mixes (Meridian Bioscience, Cincinnati, OH, USA) on a Bio‐Rad thermocycler (Bio‐Rad, Hercules, CA, USA). Standard curves for real‐time PCR assays were developed by PCR amplifying the respective genes by their specific primers, PCR products purification using a PCR cleanup kit (Axygen Bioscience, Union City, CA, USA), cloning into the pGEM‐T Easy Vector (Promega Corporation, MD, USA), and transformation into *Escherichia coli* JM109 competent cells (Promega Corporation, MD, USA) (details in Trivedi et al. 2012). Tenfold serial dilutions (10^8^–10^1^ copies per μL) of the plasmid DNA were subjected to a qPCR assay in triplicate to generate an external standard curve and to check the amplification efficiency. Standard curve regression coefficients were consistently above 0.99 and melt curve analysis verified a single amplicon per reaction in all the cases. Samples and standards were assessed in at least two different runs to confirm reproducibility of the quantification. Target copy numbers for each reaction were calculated from the standard curve and were used to ascertain the number of copies per μg of DNA.

### 2.7 In Vitro Glyphosate Assay

We used 86 bacteria previously isolated from bulk soil samples and therefore representative of the agroecosystem to evaluate the direct effect of the active ingredient glyphosate in vitro. Bacterial strains were characterised through Sanger sequencing (Eurofins Genomics, Louisville, Kentucky, USA) on the amplified 16S rRNA using primers 27f‐1492r (Kanagawa 2003). The bacteria were initially isolated on nutrient agar medium and stored as glycerol stock in the −80°C freezer. Bacterial strains were revived twice on nutrient agar from glycerol. Isolated bacterial colonies were then inoculated into fresh nutrient broth, the stock was homogenised by vortexing and adjusted to 1 ⋅ 10^8^ cells. Bacterial nutrient broth stock was added to a 1.1 mL 96‐well plate with 10 mM glyphosate dissolved in nutrient broth. Positive control wells containing bacteria without glyphosate and negative controls without glyphosate or bacteria were included on each plate. Three replications were performed for each bacterial strain. The plates were incubated at 30°C for 48 h and absorbance of the plates was read at 600 nm using a microplate reader (Tecan Infinite Pro 200, Mannedorf, Switzerland) and analysed in the Tecan iControl software (Tecan, Crailsheim, Germany). Percent growth inhibition was calculated by comparing the absorbance of bacteria exposed to glyphosate and the positive controls:

$$\%\text{Inhibition}=\frac{\mathrm{Ab}s_{\text{control}}-\mathrm{Ab}s_{\text{treatment}}}{\mathrm{Ab}s_{\text{control}}}\times 100$$

### 2.8 Statistical Analysis

For analysing amplicon sequence data, samples were rarified to the lowest occupancy of 10,000 reads. We used the R package ‘mctools’ to analyse microbial community structure (Leff 2017). To examine alpha diversity (metrics that describe the species richness, evenness, or diversity within a sample), Shannon diversity indices (alpha diversity index that quantify the diversity of a community, taking into account both the number of species [species richness] and the evenness of their distribution) was calculated. To account for non‐independence within plots and repeated measures across time, linear mixed models (LMMs) were used to assess the effects of experimental treatments on microbial diversity, abundance of functional groups or soil enzyme activities. The mixed model analysis of variance (ANOVA) was performed using the ‘lmer’ function from the lme4 package in R (Bates et al. 2015).

We also tested general linear models (GLMs) to evaluate the effects of sampling timepoints and its interaction with experimental treatments. Tukey honestly significant difference (HSD) tests were used to determine influence of the treatments on alpha‐diversity. Scatter plots were constructed in ‘ggplot2’ using Shannon's diversity metric and correlated to actual sampling date (Wickham 2016). Permutational multivariate analysis of variance (PERMANOVA) models were generated to determine significant beta‐diversity (measure of the similarity or dissimilarity of two communities) differences correlating with timepoint and treatment. Bray–Curtis dissimilarity distances were calculated then ordinated in non‐metric multidimensional scaling plot (NMDS) analysis by treatment for each timepoint. Scatter plots were further constructed to visualise temporal changes using Axis 1 from the NMDS ordination plots for each timepoint. Statistical analysis of soil enzyme activity in different treatments were analysed using a one‐way ANOVA. Scatter plots were constructed in relation to sampling date. Cycle threshold (Ct) values from qPCR data were normalised to a standard curve and calculated to gene copies per nanogram of DNA based on DNA concentration. Boxplots were constructed in ‘ggplot2’, and significance was evaluated based on Tukey's HSD (Wickham 2016). Volcano plots used to visualise significantly enriched or depleted taxa for treatments were constructed using ‘DESeq2’ (Love et al. 2014). A significance level of *α* = 0.05 was used for all biomarkers evaluated in this study. All statistical analyses were completed using R version 4.0.5 (R Core Team 2022).

The visualisation of the in vitro effects of glyphosate on the bacteria isolates was performed using the interactive tree of life (iTOL) interface (Letunic and Bork 2021). First, the bacterial phylogenetic tree was constructed using Clustal Omega by EMBL‐EBI (Madeira et al. 2022). The tree and the metadata files derived from the in vitro assays were imported into the iTOL interface. In the iTOL output, the growth for individual strains is displayed as percent growth inhibition. Significance was determined through a two‐tailed Student's *t*‐test with a significance level of *α* = 0.05.

## 3 Results and Discussion

### 3.1 Herbicide Applications Have Minimal Impact on Bacterial Community Structure

We compared the impact of tillage on the diversity and structure of soil bacterial communities through comparing the samples taken from the tillage treatment before and after each tillage event. Our results showed non‐significant differences in alpha diversity due to tillage in the Wyoming plot; however, we did observe significant differences in alpha diversity related to tillage events in the Nebraska plot (Figure S1). We observed differences in the bacterial community composition within both plots, visualised by significant clustering in the beta diversity ordinations (Figure S2). Our results are in line with previous studies showing a significant impact of tillage on the structure of soil microbial communities (Feng et al. 2003; Khan et al. 2023; Mathew et al. 2012). Due to the inconsistent results on the impact of tillage, here we focus on the impact of herbicide application on soil microbial communities. Therefore, we considered the tillage treatment as a control where no herbicide was applied.

**FIGURE 1:**
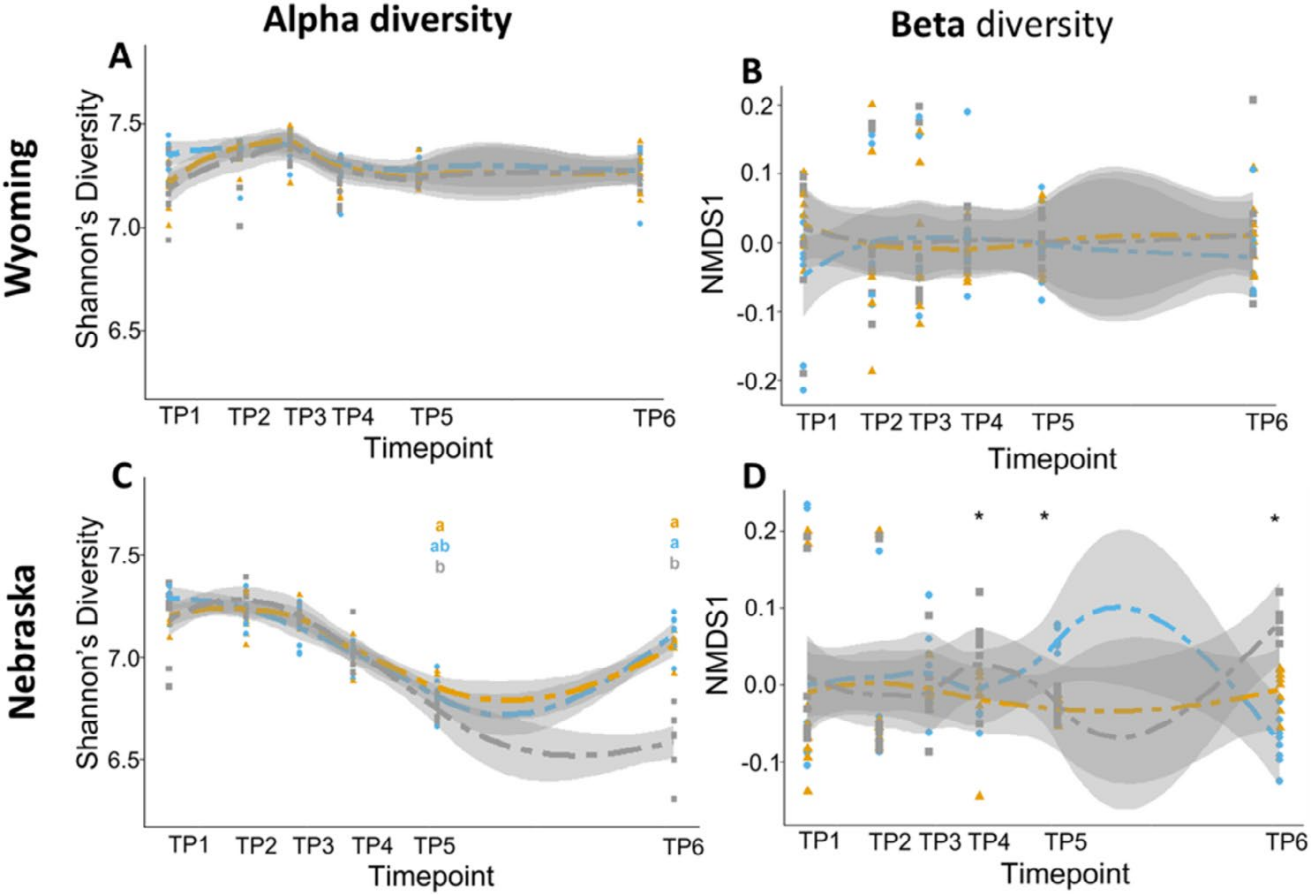
Temporal representation of the alpha diversity for the Lingle, WY site (A) and the Scottsbluff, NE site (C) is based on Shannon's diversity metric. Letters a, b and ab correspond to colour of treatment, and represent groups that are significantly different from each other based on Tukey's HSD test. Beta diversity is represented by Axis 1 of NMDS ordination plots constructed for each timepoint for the Lingle, WY site (B) and the Scottsbluff, NE site (D). Significant differences between sample communities based on PERMANOVA are represented by an asterisk (*) (Table S3). A significance level of *α* = 0.05 was used for statistical analysis. Glyphosate (blue), mixed selective herbicide (orange) and control (grey) treatments are represented as scatter plots with standard error shading. Treatment timing for the mixed selective herbicide (MS), glyphosate (G) and tillage (T) treatments are indicated in the legend. Specific application information is available in Table 1.

Our LMM models showed a non‐significant impact of herbicide‐based weed management strategies on the bacterial alpha‐diversity. The GLM analysis revealed that the differences in microbial structure and function were temporal and variable based on environment, with no consistent differences between herbicide‐based weed management. In the experimental site in Wyoming, the impact of timepoint on alpha diversity was significant (*p* < 0.001); however, there was no significant difference between the use of herbicides or tillage for weed management (Table S2). At the Nebraska site, both timepoint (*p* < 0.001) and treatment (*p* < 0.001), as well as the interaction between timepoint and treatment (*p* < 0.001), significantly impacted alpha diversity. Treatment‐mediated impacts were more apparent in the later timepoints (Figure 1). Specifically, there was a decrease in Shannon's diversity for the control plot (6.58) compared to the glyphosate and mixed selective herbicide (7.11 and 7.05, respectively) treatments. Our results showed similar trends to those reported by other studies in this area, wherein we observed high taxonomic and functional microbial diversity in all treatments, with significant differences between locations (Lupwayi et al. 2022). As for the drop in diversity following tillage in Scottsbluff, there are isolated instances of significant effects to soil microbial diversity following even single tillage events (Kraut‐Cohen et al. 2020). The question remains whether detectable differences in the alpha diversity translate to biologically relevant impacts. For example, the impacts of disturbances on soil functionality are explained by microbial community structure and biomass rather than the alpha diversity (Zhou et al. 2020).

PERMANOVA analysis was used to determine the influence of treatments on the communities over six timepoints. At the Wyoming site, PERMANOVA analysis showed no significant differences between the treatments at any timepoint. In the Nebraska site, we did not find significant differences during the first three timepoints; however, there was a significant difference in community composition between both herbicide and tillage treatments in the last three timepoints based on the PERMANOVA (Figure 1). The variation in microbial community diversity between these two sites represents the high impact of site on microbial diversity and therefore response to changes in the soil from tillage (Babujia et al. 2016; Lupwayi et al. 2022).

Both sites had a similar relative abundance at the beginning of the season; however, they diverged significantly throughout the season for all treatments. Proteobacteria (25%–30%) was the most dominant phylum in relative abundance, followed by Bacteroidetes (21%–22%) and then Actinobacteria (11%–13%) at both sites for all treatments at the first timepoint (Figure S3). While we observed temporal changes in microbial relative abundance throughout our sampling period, the treatments had little impact. We did not observe any depletion or enrichment of taxa for either following herbicide treatment when compared to tillage alone at the Wyoming site over the sampling period, as visualised in volcano plots (Figure S4). In Nebraska, there was a transient reduction of Cyanobacteria following all herbicide treatments. However, Cyanobacteria only accounted for 1.05% of the total relative abundance of species across the three treatments. The temporal nature and low overall abundance suggest little biological relevance in this observation, as overall function, discussed later, is not impacted by these minor fluctuations. In the glyphosate treatment, there was a depletion in a few OTUs of Proteobacteria (3 OTUs), Bacteroidetes (2 OTUs), Fibrobacteria (1 OTU) and Firmicutes (1 OTU), while in our mixed selective herbicide plot, we observed a depletion in Firmicutes (1 OTU) and an enrichment in Bacteroidetes (1 OTU) compared to tillage. The vast majority of these OTUs were not significantly affected by the treatments; therefore, we cannot attribute community differences to herbicide application.

While differences in soil community composition have been reported in short‐term or in vitro studies (Boldt and Jacobsen 1998; Kepler et al. 2020; Rose et al. 2016), it is important to account for all variables that could be influencing the results. Short‐term or single timepoint field studies have also observed significant differences due to herbicide inputs (Arango et al. 2014). However, our study has demonstrated that the temporal and environmental influence is the greatest driver of differences compared to herbicide treatment. Consistent with our study, long‐term field studies examining the impact of herbicide inputs on the microbiome have observed few negative impacts on species richness and biomass (Aguiar et al. 2020; Busse et al. 2001; Cheng et al. 2008; Kepler et al. 2020; Schlatter et al. 2017; Weaver et al. 2007).

### 3.2 Function of Bacterial Communities Are Not Impacted by Herbicide Treatments

**FIGURE 2:**
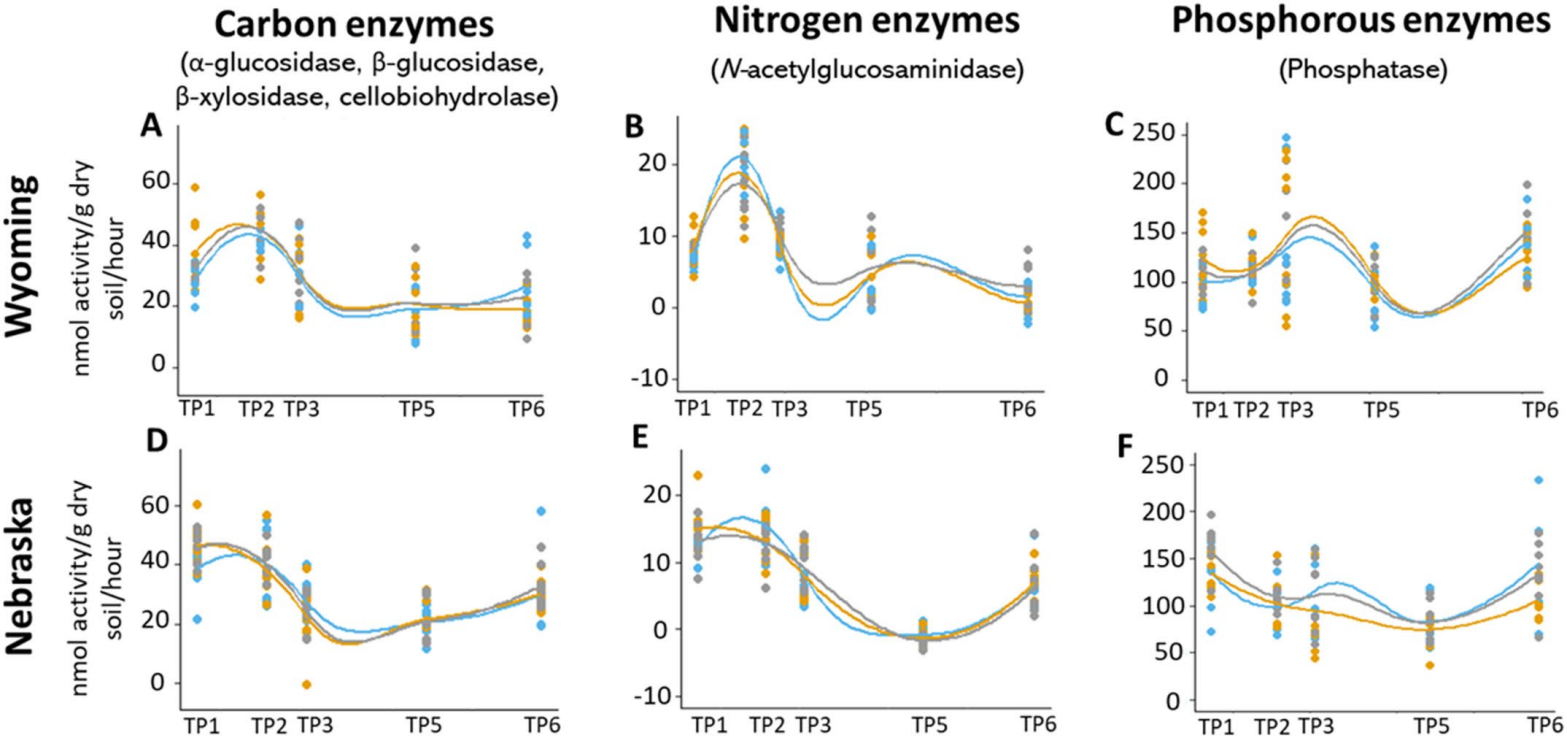
Extracellular enzyme activities at five timepoints for the Wyoming (A–C) and Nebraska (D–F) sites are normalised to nmol/g dry soil/h. Carbon enzymes represent an average of α‐glucosidase, β‐glucosidase, β‐xylosidase and cellobiohydrolase production, *N*‐acetylglucosaminidase production represents relative nitrogen cycling and phosphatase represents phosphorous cycling. Glyphosate (blue), mixed selective herbicide (orange) and tillage (grey) treatments are represented as scatter plots. No significant differences are observed between the treatments throughout the timepoints.

Nutrient cycling by microbes can improve plant growth, resistance to disease, and decrease dependency on synthetic fertilisers; therefore, it is essential to understand the impacts of herbicide application and tillage on the functional ability of the microbiome. Microbial extracellular enzyme production can be regulated by nutrient presence in the soil and is a good indicator of nutrient cycling and soil health (Luo et al. 2017). The LMM approach revealed no significant difference between treatments for enzyme activity for both sites. However, all treatments experienced similar temporal changes (Figure 2). Temporal changes in enzyme activity throughout the season are expected due to variation in nutrient cycling from plants at different growth stages as well as crop residue (Jat et al. 2019; Mandal et al. 2007). These results are consistent with a study by Tyler (2022), where only minor differences were observed in BG, CB, NAG, and PHOS enzyme activity over 2 years after the application of glyphosate and 2,4‐D. Other studies have had variable, fluctuating results based on active ingredient and rate of application of herbicide (Lupwayi et al. 2022; Z. Du et al. 2018). Our results demonstrate that enzyme function was not affected by herbicide application, which can give insight into the impacts of this management strategy on overall soil health.

At field application rates, soil respiration was reported to be stimulated with the application of glyphosate (Lane et al. 2012; Means et al. 2007) or unchanged (Pereira et al. 2008; Zabaloy and Gómez 2008), with limited studies seeing a reduction in microbial respiration compared to non‐treated sites. Decrease in microbial respiration could be plant‐mediated due to less plant species present at treatment sites, rather than a direct effect of the herbicide (Rose et al. 2016). There were no significant differences in microbial respiration between the glyphosate, mixed selective, and control treatments (Figure S5).

**FIGURE 3:**
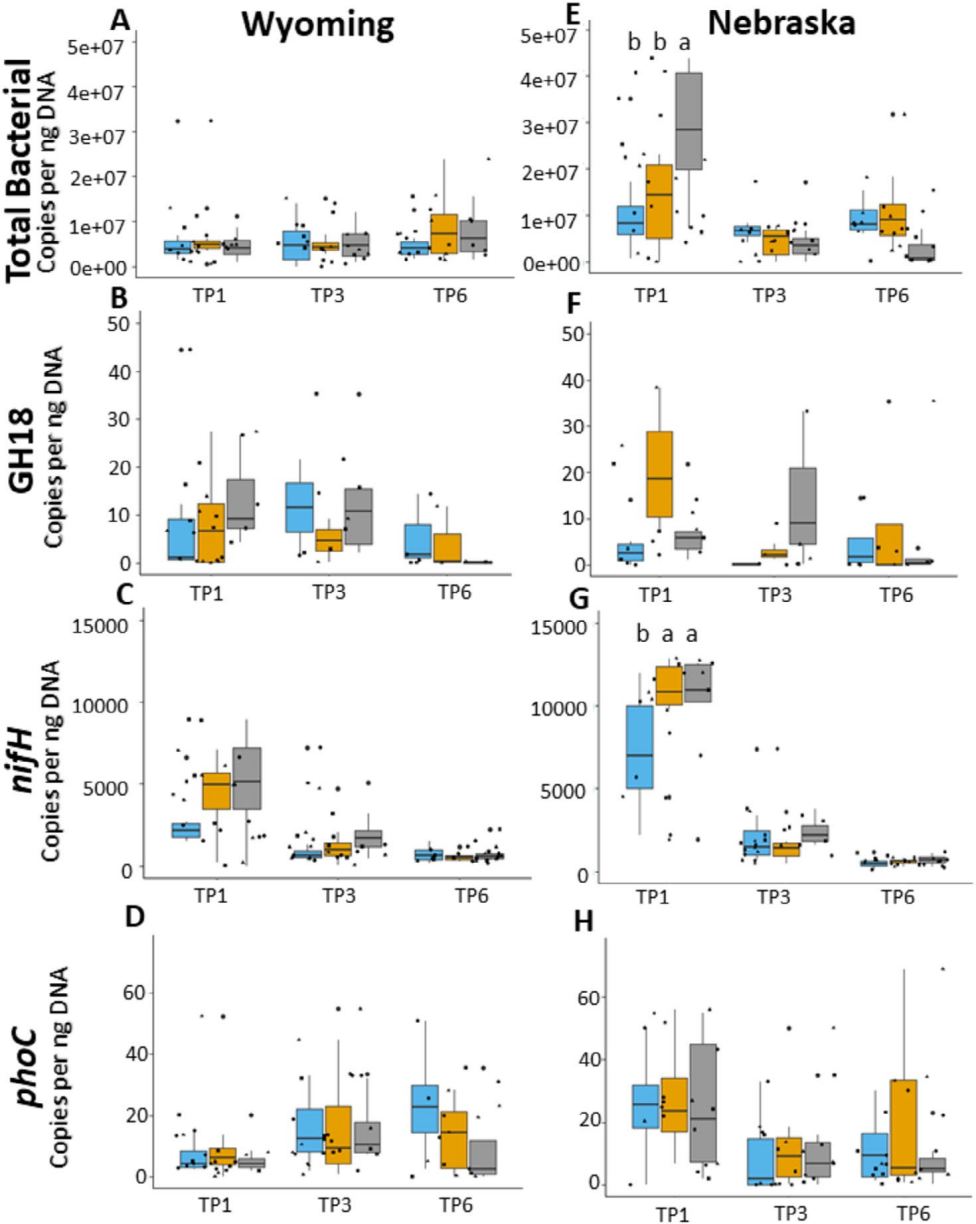
Quantitative PCR (qPCR) data demonstrating functional gene abundance in soil samples for total bacterial genes (A, E), carbon cycling gene GH18 (B, F), nitrogen cycling gene NifH (C, G) and phosphorous cycling gene PhoC (D, H) for the Wyoming (A–D) and Nebraska (E–H) plots at the first, third and sixth sampling timepoints. Ct values from qPCR were normalised to a standard curve and expressed as copies per ng of DNA. Glyphosate (blue), mixed selective herbicide (orange) and control (grey) treatments are represented as box and whiskers plots.

Functional gene abundance in the soil is another indicator of soil health. Overall, there were no significant differences in functional gene abundance related to herbicide treatment (Figure 3). Few differences were observed in the first timepoint in the Nebraska site; however, those changes disappeared later in the season. Ribosomal gene quantification for total bacteria revealed an increase of gene copies per ng of DNA in the tillage treatment compared to the two herbicide treatments. Within the nitrogen cycling genes, *nifH*, *nirS*, *nirK*, and *nosZ*, only *nifH* showed significant differences between treatments at the first timepoint, with an increase in *nifH* in the glyphosate treatment plots compared to the mixed selective and tillage plots (Figure S6). Soil samples collected at the first timepoint were only impacted by the pre‐emergence application in the mixed selective treatment plots. Consequently, the differences between total bacteria and *nifH* gene abundance are not attributed to treatment at the first sampling timepoint. There were no significant differences between treatments or timepoints for *PhoC*, a phosphorous cycling gene, or GH11 and GH18, functional genes involved in carbon cycling. Like extracellular enzyme production, previous studies involving functional gene presence are highly variable and dependent on experimental design. One study observed that *nifH* is negatively correlated to herbicide residue, while *nirK* and *nosZ* were positively or not affected; however, these results were compound dependent (Walder et al. 2022). From our exploration into functional gene presence, there is minimal evidence that these herbicides are affecting soil function over a cropping season.

Like community composition, previous studies on the effect of herbicides and tillage on soil function reported high variability and inconsistency in aspects such as herbicide rates, formulations, environmental or soil conditions (Nguyen et al. 2016), as well as tillage intensity, depth, and frequency, and whether tillage is combined with added manure or compost (Chaudhry et al. 2012; Hartman et al. 2018; Kraut‐Cohen et al. 2020; Nikitin et al. 2020). Studies observing changes in microbial function due to herbicide applications have been attributed to the differences in environmental conditions (Means et al. 2007), soil organic matter (Haney et al. 2002; Tyler 2022), soil moisture (Caggìa et al. 2023) or changes in plant‐stimulated nutrient cycling (Damin et al. 2012) rather than the direct impact of the herbicide itself. One study found that the influence of glyphosate and atrazine on nutrient cycling and mineralisation was rate‐dependent; however, activity was stimulated at all rates, rather than hindered (Haney et al. 2002). Studies observing changes in microbial function due to tillage are largely related to the physical impacts of the tillage leading to less diversity and soil function as the soil becomes less stable with lower water holding capacity (Nunes et al. 2020; Zuber and Villamil 2016). Nutrient cycling can also be relative to dead plant litter and organic matter on the soil surface after application that may be absent in mechanical weeding practices (Jat et al. 2019; Rose et al. 2016).

### 3.3 In Vitro Analysis of Glyphosate Impacts Does Not Reflect Dynamics in the Agroecosystem

**FIGURE 4:**
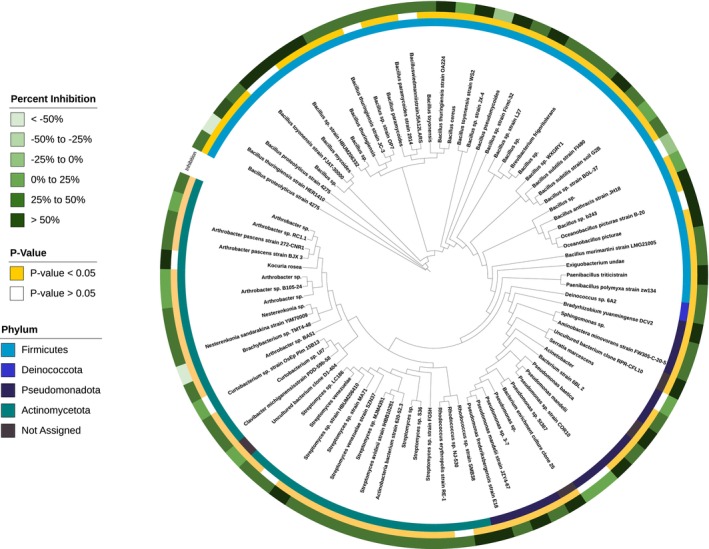
Phylogenetic tree of 86 bacteria isolated from soils representing phylum (inner circle), percent growth inhibition due to direct glyphosate exposure (centre circle) and significance (outer circle) based on a Student's *t*‐test and 0.05 significance level.

Much of the dialogue around the deleterious effect of herbicides on microbes comes from demonstrated impacts in an artificial in vitro system (Chávez‐Ortiz et al. 2022; Sharma and Khanna 2011; Lone et al. 2014; Wicke et al. 2019). In this study, a comparison between in vitro assay results and microbial structure and function under field conditions showed the lack of relevance of these types of in vitro assays (Figure 4). Out of the 86 isolates used in the assay, 69 had significantly reduced growth when cultured in the presence of glyphosate, while the growth of three isolates increased significantly. There were no observable trends based on phylogeny in the species tested. Variability in growth would be expected since bacteria can carry natural resistance to glyphosate (Motta et al. 2018) and use glyphosate as a nutrient source (Motta et al. 2018) (Jacob et al. 1988; Lerbs et al. 1990; Panettieri et al. 2013; Partoazar et al. 2011). Furthermore, the rate used in this experiment (10 mM), like many other in vitro analyses, is magnitudes higher than field‐relevant application rates. Under field conditions, direct exposure is extremely limited as it is metabolised by plants in the field, adsorbed to soil particles (Duke and Powles 2008) and interacts with diverse communities of microbes with varying metabolic interactions with the herbicide (Busse et al. 2001; Duke and Powles 2008; Lupwayi et al. 2022; Singh et al. 2020). Since herbicides can act as a source of nutrients while simultaneously being potentially toxic to susceptible bacteria, these contrasting effects could lead to limited influences on the overall microbiome in the field (Busse et al. 2001; Lupwayi et al. 2022). This study indicates the importance of a diverse, cooperative microbiome in field soils that could improve the resilience of the community that cannot be achieved in a laboratory setting. The greatest limitation of in vitro studies is the inability to compare to the only other viable non‐chemical alternative for weed control, tillage. Tillage has been widely reported to impact the health, diversity, and function of the soil microbiome, but it also has negative impacts on the physical properties of the soil critical to support sustainable agricultural production (Kraut‐Cohen et al. 2020; Nunes et al. 2020; Zuber and Villamil 2016).

## 4 Conclusion

Overall, our results showed that the relationship between microbes and herbicides and tillage is complex, and the findings related to the effect of herbicides and tillage on the soil microbial community are inconclusive. Our study highlights there is no discernible impact on microbial structure between the three weed management approaches. As with other studies of this nature, variability is based on site, and while there are minor changes in diversity, these changes are not affecting the overall function of the soil microbiome. Furthermore, this study further confirms in vitro analysis is unrepresentative of impacts within the agroecosystem. The comparison between the results of the in vitro and field analyses in this study highlights the complexity of the agroecosystem and the necessity to practise great care in experimental design meant to inform real‐world impacts. Overall, we need more well‐replicated, field‐realistic, and long‐term experiments using various commercial formulants of active ingredients and intensity of tillage to understand herbicides' and tillage's ecological and evolutionary consequences in agroecosystems. Obtaining greater information about the impacts of herbicides versus non‐chemical alternatives (e.g., tillage) on the biological diversity and function of the soil as well as the overall soil chemical and physical properties will inform future regulation and farmer decision‐making.

## Author Contributions

Pankaj Trivedi conceived and supervised the study. Pankaj Trivedi, Rebecca Larson, Franck E. Dayan, Dave Reichert, and Andrew R. Kniss designed the experiments. Abigail Thompson, Dave Reichert, Kristen Otto, Andrew R. Kniss, and Margaret Gaylord performed the experiments. Margaret Gaylord and Pankaj Trivedi analysed the data. Margaret Gaylord and Pankaj Trivedi wrote the manuscript. All authors read, edited, and approved the final manuscript.

## Conflicts of Interest

Margaret Gaylord, Abigail Thompson, Franck E. Dayan, Andrew R. Kniss, Kristen Otto, and Pankaj Trivedi declare no conflicts of interest. Rebecca Larson and Dave Reichert are employees of Western Sugar Cooperative who funded the project.

## Supporting information


**Data S1.** Supporting Information.

## Data Availability

The data that support the findings of this study are openly available in NCBI Sequence Read Archive at https://www.ncbi.nlm.nih.gov/sra, reference number PRJNA1008186.
