# Supplementary material for: Herbicides Have Minimal and Variable Effects on the Structure and Function of Bacterial Communities in Agricultural Soils
Source: Environ Microbiol. 2025 Jul 14;27(7):e70148. doi: 10.1111/1462-2920.70148 (PMC12260311; doi:10.1111/1462-2920.70148)
Supplement: Supplementary file 1 — Data S1. Supporting Information. [file EMI-27-e70148-s001.docx]

**Supplementary Information:**

**Supplementary Table 1:** Differences in the soil properties between Wyoming and Nebraska sites.

| Site | Soil pH | Organic Matter LOI % | Nitrate-N ppm N | Potassium ppm K | Sulfate-S ppm S | Calcium ppm Ca | Magnesium ppm Mg | Sodium ppm Na | Mehlich P-III ppm P |
| --- | --- | --- | --- | --- | --- | --- | --- | --- | --- |
| Wyoming | 8.4 | 0.8 | 15.8 | 279 | 9.3 | 4213 | 96 | 11 | 34 |
| Nebraska | 8 | 3.9 | 30.7 | 486 | 100 | 4716 | 442 | 110 | 97 |

**Supplementary Table 2.** The effects of timepoint, treatment and their interaction on the alpha diversity of samples within the two experimental sites based on ANOVA.

|  | **Wyoming** | | | **Nebraska** | | |
| --- | --- | --- | --- | --- | --- | --- |
| Drivers | Sum of Squares | F-value | P-value | Sum of Squares | F-value | P-value |
| Timepoint | 0.49469 | 11.3734 | 4.55E-09 | 3.7549 | 90.31 | 2.20E-16 |
| Treatment | 0.07321 | 4.208 | 0.01702 | 0.2679 | 16.105 | 6.13E-07 |
| Timepoint:Treatment | 0.11298 | 1.2987 | 0.23818 | 1.2388 | 14.886 | 2.20E-16 |

**Supplementary Table 3.** PERMANOVA results showing the impact of treatment on the soil bacterial communities at each timepoint.

| **Wyoming** | | | **Nebraska** | | |
| --- | --- | --- | --- | --- | --- |
| Timepoint 1 | | | | | |
| R^2^ | F-value | P-value | R^2^ | F-value | P-value |
| 0.0946 | 1.1 | 0.215 | 0.105 | 1.23 | 0.143 |
| Timepoint 2 | | | | | |
| R^2^ | F-value | P-value | R^2^ | F-value | P-value |
| 0.0704 | 0.796 | 0.951 | 0.0708 | 0.801 | 0.862 |
| Timepoint 3 | | | | | |
| R^2^ | F-value | P-value | R^2^ | F-value | P-value |
| 0.0708 | 0.80 | 0.971 | 0.094 | 1.04 | 0.25 |
| Timepoint 4 | | | | | |
| R^2^ | F-value | P-value | R^2^ | F-value | P-value |
| 0.0891 | 1.03 | 0.308 | 0.188 | 2.21 | 0.001* |
| Timepoint 5 | | | | | |
| R^2^ | F-value | P-value | R^2^ | F-value | P-value |
| 0.0803 | 0.917 | 0.922 | 0.198 | 2.59 | 0.001* |
| Timepoint 6 | | | | | |
| R^2^ | F-value | P-value | R^2^ | F-value | P-value |
| 0.0862 | 0.991 | 0.492 | 0.269 | 3.85 | 0.001* |


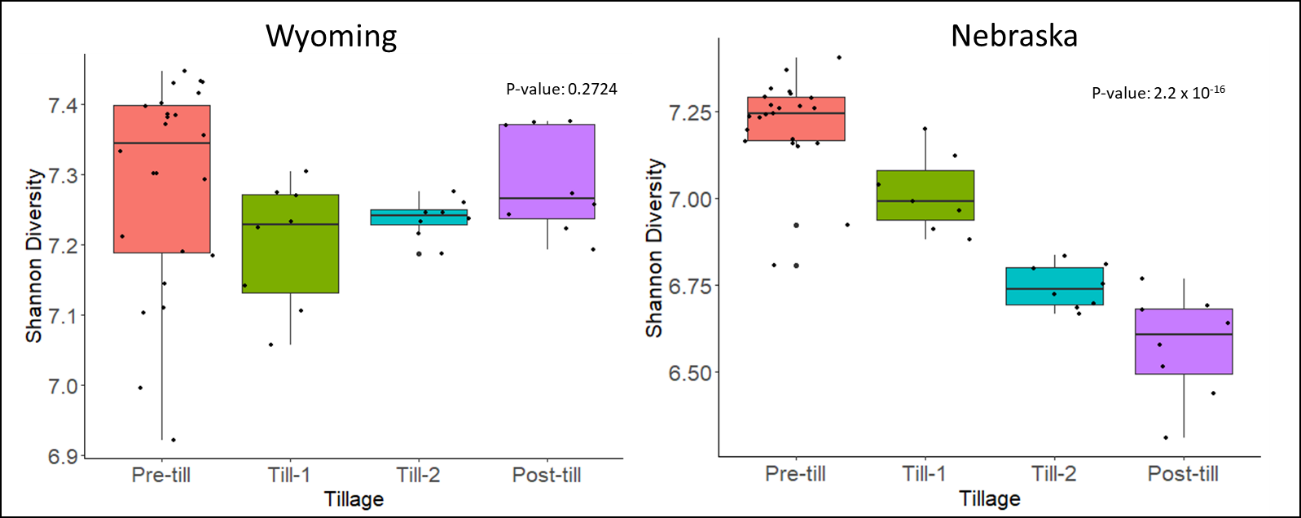


**Supplementary Figure 1.** Alpha diversity from the Wyoming (left) and Nebraska (right) plots for the tillage treatment. Groups are represented by pre-tillage (timepoint 1-3), immediately after the first tillage event (timepoint 4), immediately after the second tillage event (timepoint 5), and approximately one month after the second tillage event (timepoint 6). P-value is based on an ANOVA with a significance level of 0.05.


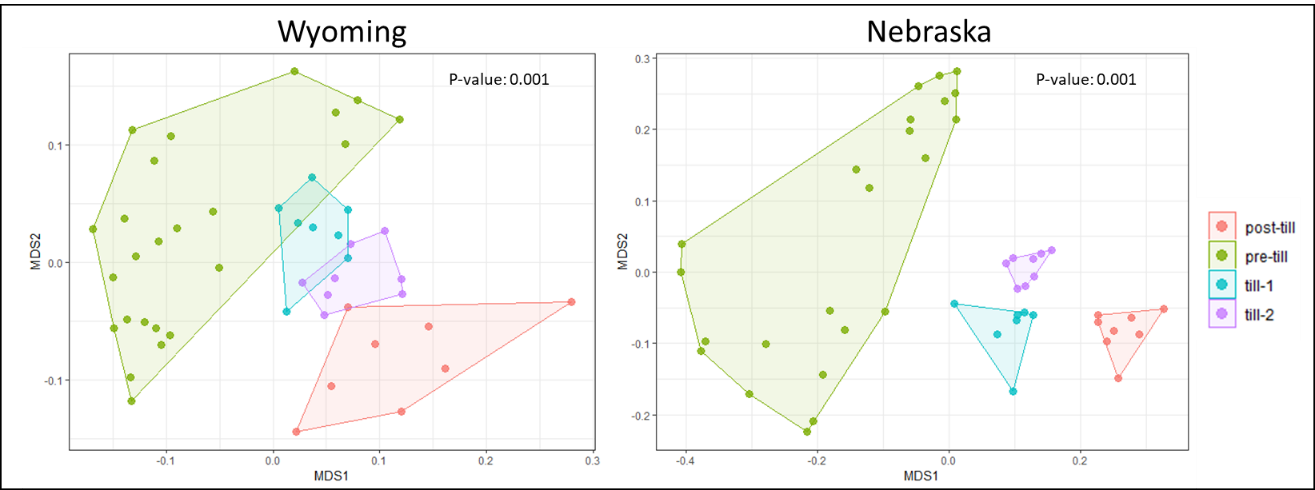


**Supplementary Figure 2.** NMDS ordination plot representing differences in beta diversity between samples from the tillage treatment. Groups are represented by pre-tillage (timepoint 1-3), immediately after the first tillage event (timepoint 4), immediately after the second tillage event (timepoint 5), and approximately one month after the second tillage event (timepoint 6). P-value is based on a PERMANOVA with a significance level of 0.05.


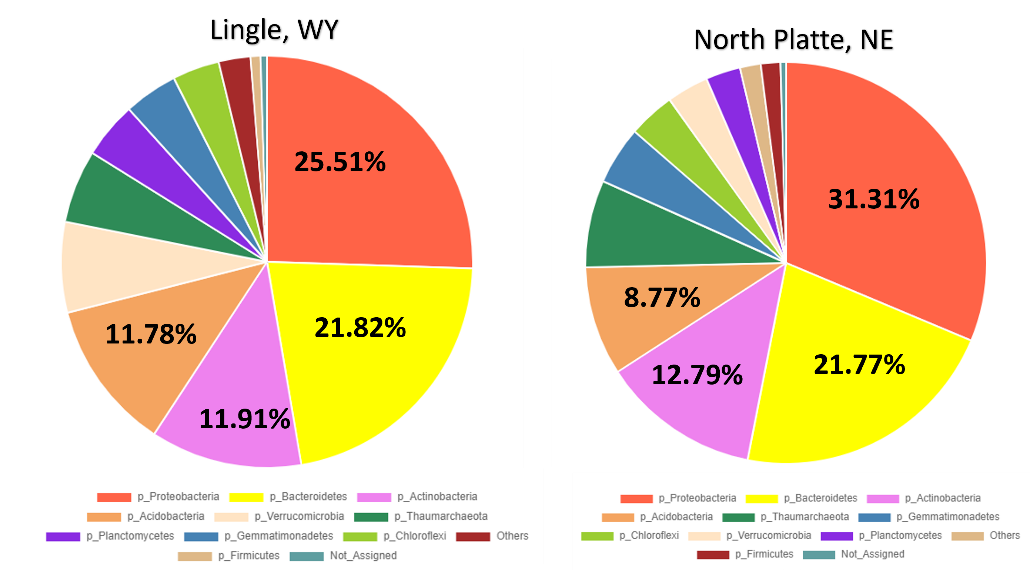


**Supplementary Figure 3.** Pie chart representing the percent relative abundance of each phylum for the combined treatments plots at the first sampling point at Lingle, WY (left) and North Platte, NE (right) sites.


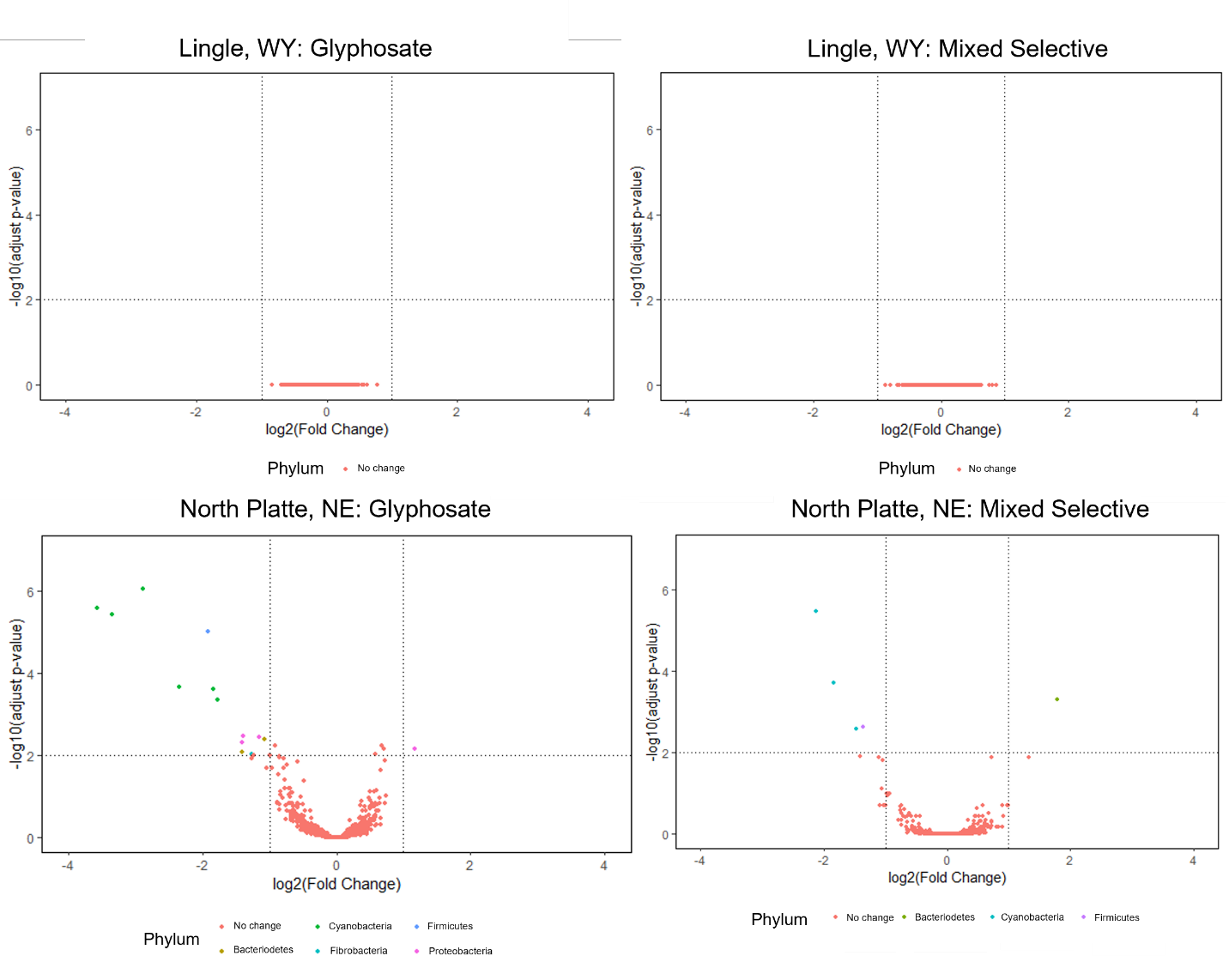


**Supplementary Figure 4.** Volcano plots representing the total enriched (right side) and depleted (left side) OTUs for the Wyoming (top) and Nebraska (bottom) sites.


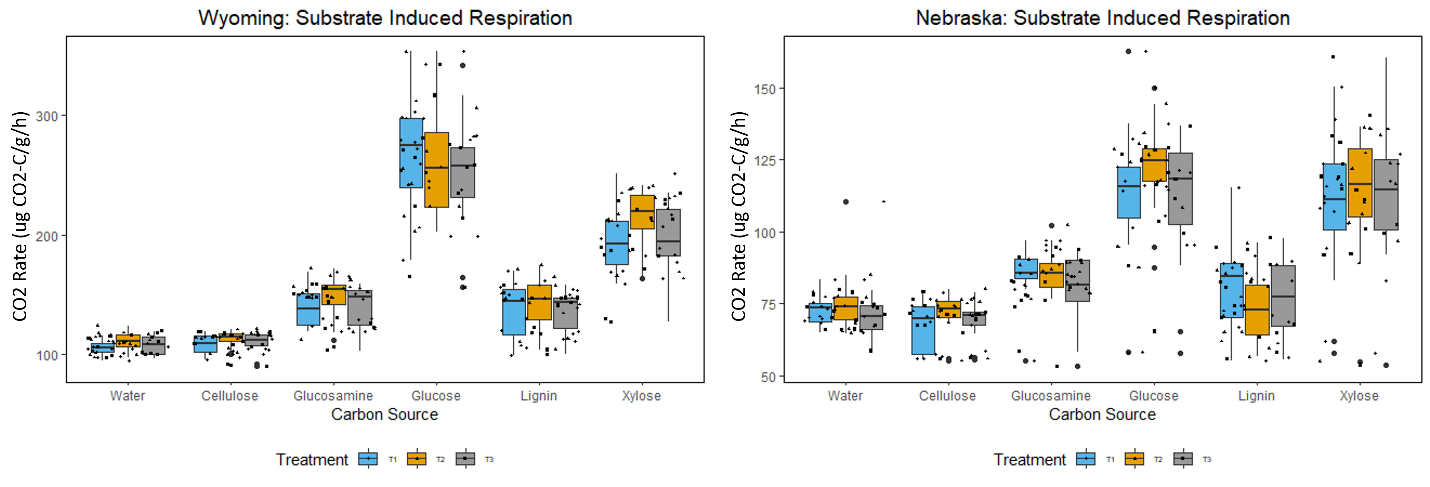


**Supplementary Figure 5.** Carbon substrate induced microbial respiration for the Lingle, Wyoming (left) and North Platte, Nebraska (right) plots at the third sampling timepoint.


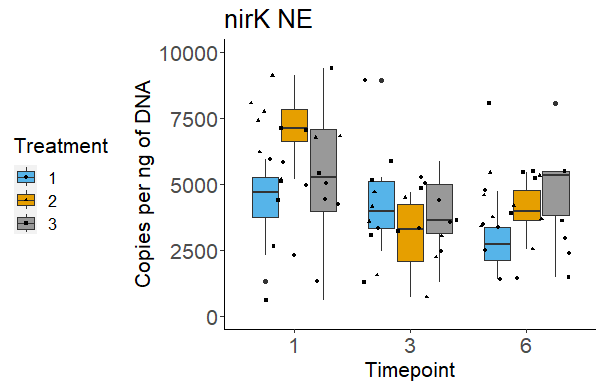

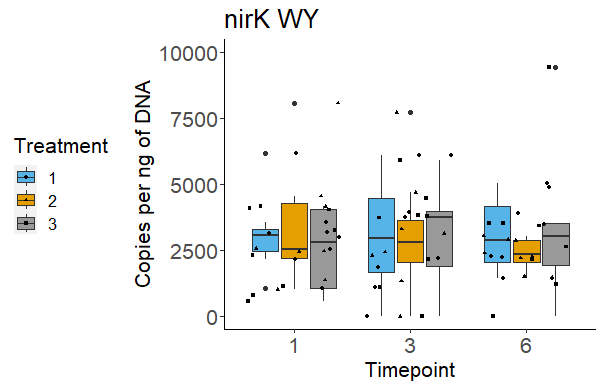

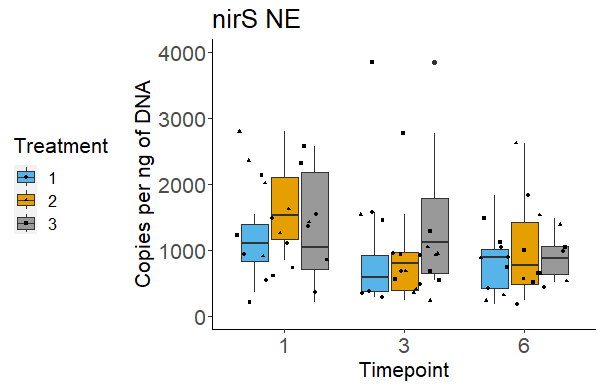

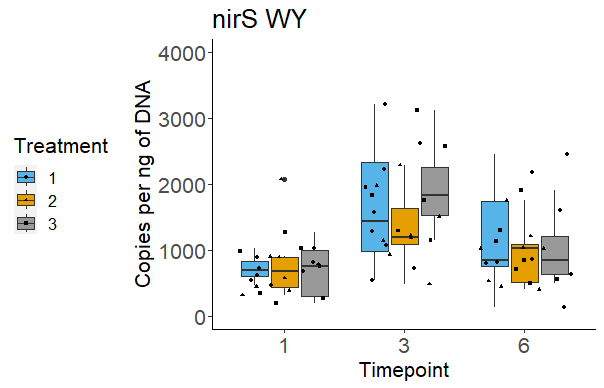

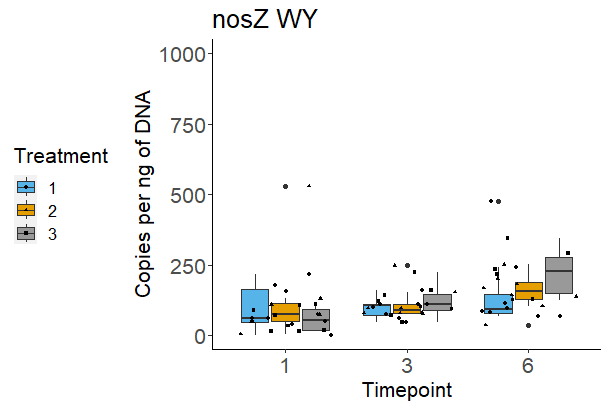

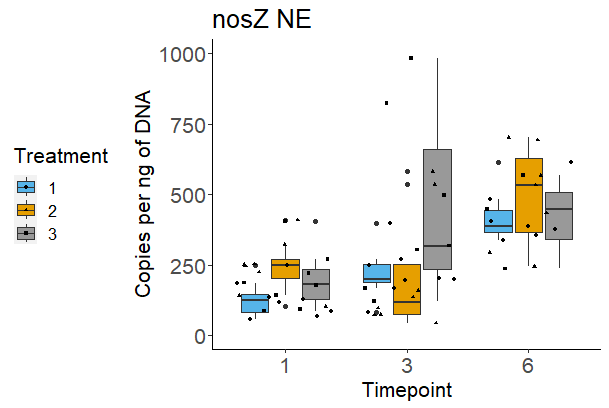

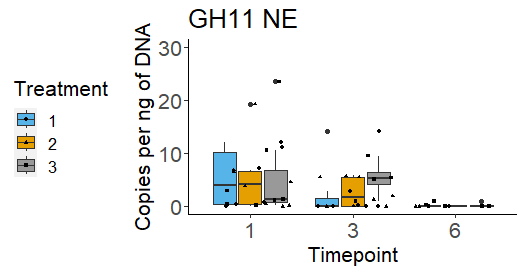

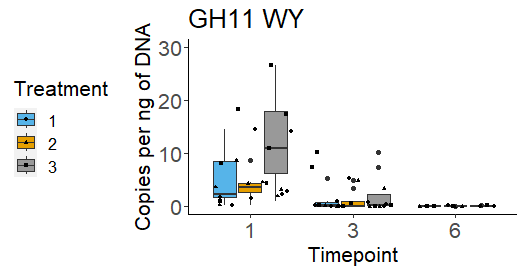

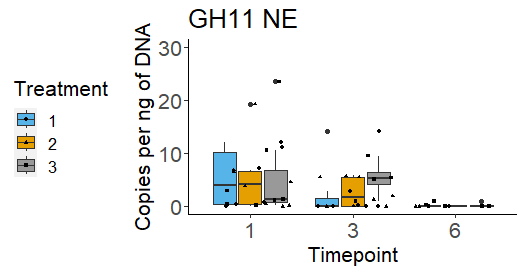


**Supplementary Figure 6.** Quantitative PCR data demonstrating functional gene presence in soil samples for *nosZ*, *nirK*, *nirS* and GH18 at timepoints 1, 3 and 6 for Lingle, Wyoming (top) and North Platte, Nebraska (bottom).
